# Introduction of Two-Dose Mumps-Containing Vaccine into Routine Immunization Schedule in Quzhou, China, Using Cox-Proportional Hazard Model

**DOI:** 10.1155/2021/5990417

**Published:** 2021-11-05

**Authors:** Zhiying Yin, Canjie Zheng, Quanjun Fang, Xiaoying Gong, Guoping Cao, Junji Li, Ziling Xiang, Wei Song

**Affiliations:** ^1^Quzhou Center for Disease Control and Prevention, Quzhou, 324000 Zhejiang, China; ^2^Quzhou Women & Children's Hospital, Quzhou, 324000 Zhejiang, China

## Abstract

Mumps is a vaccine-preventable disease caused by the mumps virus, but the incidence of mumps has increased among the children who were vaccinated with one-dose measles-mumps-rubella (MMR) in recent years. In this study, we analyzed the influence of different doses of mumps-containing vaccine (MuCV) against mumps using Cox-proportional hazard model. We collected 909 mumps cases of children who were born from 2006 to 2010 and vaccinated with different doses of MuCV in Quzhou during 2006-2018, which were all clinically diagnosed. Kaplan-Meier survival methods and Cox-proportional hazard model were used to estimate the hazard probabilities. Kaplan–Meier curves showed that the cumulative hazard of male and female has no difference; lower hazards were detected among those who were vaccinated with two-dose MuCV, born in 2006, and infected after supplementary immunization activities (SIA). Cox-proportional hazard regression suggested that onset after SIA, born in 2006, and vaccinated with two-dose MuCV were protective factors against infection even after adjusting for potential confounding effects. Our study showed that it was necessary to revise the diagnostic criteria of mumps and identify RT-PCR as the standard for mumps diagnosis in China. We suggested that routine immunization schedule should introduce two doses of MMR and prevaccination screening should be performed before booster immunization in vaccinated populations.

## 1. Introduction

Mumps is a common childhood viral disease caused by the mumps virus, and the most common symptom manifests as swelling of the parotid or other salivary glands [1]. In China, mumps was classified as a category C notifiable communicable disease in 1990 and was mandatorily registered in the Chinese Information System for Disease Control and Prevention (CISDCP) since 2004, a web-based computerized reporting system. Mumps is a vaccine-preventable disease. Routine vaccination has been proven to be highly effective in reducing the incidence of mumps. However, the incidence of mumps has increased in recent years. From 2005 to 2014, 115,745 mumps cases were reported in Shandong [2]; the incidence of children aged 0–14 years in Zhejiang Province from 2008 to 2017 was 16.88 per 100,000 [3]. Ongoing mumps outbreaks [4, 5] suggested that current immunization schedule can be improved to be adapted to disease control.

Vaccination with mumps-containing vaccine (MuCV) was the best way to prevent mumps infection [6]. Mumps vaccination was initiated in Quzhou since 1998, including the monovalent mumps vaccine (S79 strain) and the measles-mumps-rubella (MMR) vaccine developed by Merck (Jery1-Lynn vaccine strain). MuCV was a nonimmunization program vaccine, which parents had to pay out-of-pocket by themselves. In 2007, domestic MMR (S79 strain) was introduced into the Expanded Program on Immunization (EPI) for children who were born after the 1st January 2006, and replaced the second routine dose of measles vaccine for children 18 to 24 months old. However, there was an outbreak of mumps in 2009 in Quzhou, and the reported incidence was 73.91 per 100,000 of the population [7]. In order to control mumps and speed up the process of measles elimination in 2012, supplementary immunization activities (SIA) using measles mumps (MM) throughout Zhejiang Province were performed during September to December in 2010. The target populations were the children born from 1 October 2005 to 31 December 2009, who received one dose of MM free of charge, whether they were local or mobile children, and with or without a history of MuCV. The remaining MM of SIA was used for routine immunization. The children born from 2006 to 2010 can be vaccinated with different doses of MuCV. In this study, we analyzed the influence of vaccination against mumps using Cox-proportional hazard model and put forward some measures to control mumps in Quzhou, China.

## 2. Material and Methods

### 2.1. Setting

Quzhou is a prefecture-level city located in Zhejiang Province in eastern China and covers an area of 8,844 square kilometers. By the end of 2020, the total population of Quzhou amounted to 2.58 million and the birth rate was approximately 9.3 per 1,000. Quzhou lies at the junction of Fujian, Zhejiang, Jiangxi, and Anhui provinces, which means Quzhou is a major transportation hub and enjoys a convenient transportation by air, water, rail, and road. Convenient transportation networks play an important role of the rapid spread of infectious diseases. Quzhou consists of 6 districts and 2 of which are classified as urban areas; the others are considered rural. Quzhou has 108 immunization clinics, which take charge of vaccinating all children residing in the local areas, regardless of whether they were locally born or migrated. Since 2005, all vaccination information of children aged less than 15 years in Quzhou were registered in the Zhejiang Provincial Immunization Information System (ZJIIS). Each person in ZJIIS is assigned a unique identification number when they first contact the immunization clinic, which corresponds to the child's demographic information, historical immunization data, and current immunization status.

### 2.2. Case Definition

The mumps cases were diagnosed according to the diagnostic criteria for mumps approved by the Ministry of Health of China in 2007 [8]. A clinical case of mumps was defined as a person of acute onset of unilateral or bilateral swelling of the parotid gland or other salivary glands characterized by any of the following, which could not be explained by another more likely diagnosis: (1) fever, headache, weakness, and loss of appetite; (2) orchitis; (3) pancreatitis; and (4) encephalitis and/or aseptic meningitis. A laboratory-confirmed case was defined as a clinical case with one of the following laboratory evidences: (1) positive for IgM antibodies and no mumps vaccine within one month; (2) the titer of double serum IgG increased by 4 times or more than 4 times; and (3) isolated mumps virus in throat swab, urine, or brain crest fluid.

### 2.3. Data Resources

The mumps cases were obtained from the CISDCP, which requires that all cases of mumps that were clinically diagnosed or laboratory-confirmed must be reported within 24 hours since 2004. Because of the difficulty of testing in the laboratory, all mumps cases in our study were clinically diagnosed cases. Basic information of mumps cases of the children born from 2006 to 2010 were obtained from the CISDCP on December 31th, 2018, including name, name of father or mother, gender, date of birth, phone number, group classification, current address, date of disease onset, date of diagnosis, date of report, and case classification (clinical or laboratory-confirmed). Cases were matched based on name, gender, date of birth, and name of father or mother in ZJIIS in order to get their historical immunization data. Other cases that did not match in ZJIIS were investigated by telephone or face to face.

921 mumps cases who were born from 1 January 2006 to 31 December 2010 were reported via the CISDCP in Quzhou during 2006-2018. 858 (93.16%) were matched in ZJIIS, 51 (5.54%) were investigated by telephone or face to face, 10 (1.08%) were repeated, and 2 (0.22%) could not be contacted or left Quzhou. Finally, 909 mumps cases were included in this study.

### 2.4. Statistical Analysis

Descriptive statistics such as rate and proportion were used to summarize the sample characteristics; count data among cases was analyzed by the chi-square test. Cochran's and Mantel-Haenszel (CMH) test and odds ratio (OR) with 95% confidence intervals (CI) were used to analyze the influence of birth cohort to mumps incidence between before and after SIA. Month of disease onset among cases with different-dose MuCV was analyzed by the ANOVA test. The Kaplan–Meier method was used to estimate the cumulative hazard probabilities of mumps with gender, dose of MuCV, year of birth, and onset before or after SIA, and the differences were assessed by the log-rank test. Hazard ratios (HR) with 95% confidence intervals (CI) and the cumulative hazard with different doses were estimated by Cox-proportional hazard regression analysis. We performed all analysis with statistics software SPSS 16.0 (SPSS Inc., Chicago, Illinois, USA) and at a significance level of 0.05.

### 2.5. Ethical Considerations

Strict regulations were established and supervised by the China CDC to protect patients' privacy. The local CDCs were given access to the surveillance data for the purpose of research. Personal data was anonymized by deleting the personal identifiers (such as patient name, address, and telephone number) and determined as exempt from ethical review by the ethics committee of Quzhou Centers for Disease Control and Prevention (QZCDC).

## 3. Results

### 3.1. General Characteristics of the Cases

The proportion of 909 mumps cases before SIA was 17.93%, and it was 82.07% after SIA. There was no significant difference about the gender of the cases between before and after SIA. Before SIA, no children were vaccinated with two-dose MuCV. Compared to the case before SIA, the incidence increased among kindergarten children and students, but it decreased among scattered children. The mumps incidence before and after SIA was 54.39 (163/2.99698) and 76.07 (746/9.806745) per 100,000 person-years, respectively (*χ*^2^ = 15.21, *p* < 0.001). Except the factor of birth cohort, the mumps incidence after SIA was 1.56 (1/0.642) times of that before SIA (Table 1).

### 3.2. Immunization Characteristics of MuCV

Of 909 cases, 8.14% were cases without MuCV, 38.94% were cases with one-dose MuCV, and 52.92% were cases with two-dose MuCV. There was no difference in the sex ratio among the three groups. The mean age of mumps cases was 40 months, 54 months, and 81 months among the different-dose MuCV group, respectively. The main cases occurred in groups of scattered children, kindergarten children with one-dose MuCV, and students with two doses of MuCV. There was no significant difference among the cases without MuCV in the 2006-2010 birth cohorts (*χ*^2^ = 8.22, *p* = 0.084). Except for the birth cohort in 2010, the proportion of cases receiving one-dose MuCV vaccination has dropped from 41.78% for 2006 birth cohort to 25.33% for 2009 birth cohort, while the proportion of cases who were vaccinated with two-dose MuCV increased from 48.89% for the 2006 birth cohort to 72.00% for the 2009 birth cohort (Table 2).

### 3.3. The Cumulative Hazard of Mumps Infection

**Figure 1:**
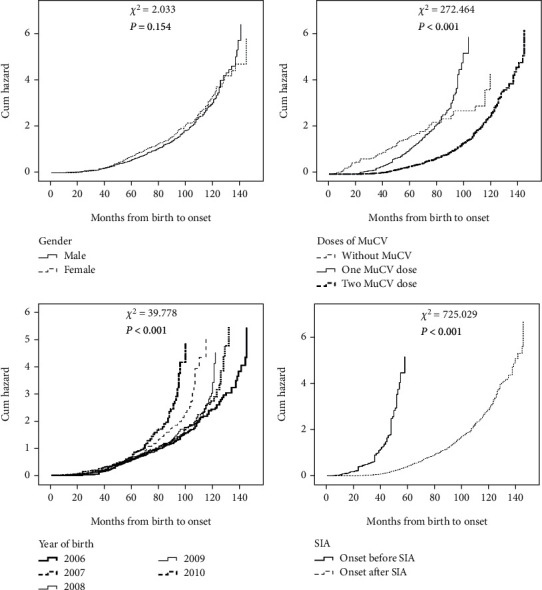
The Kaplan-Meier curves showing the cumulative hazard of mumps.

Kaplan–Meier curves showed the cumulative hazard of mumps infection by gender, doses of MuCV, year of birth, and before and after SIA. There was no difference in cumulative hazard between men and women. Lower hazards were detected among those who were vaccinated with two-dose MuCV, born in 2006, and infected after SIA (Figure 1).

**Figure 2:**
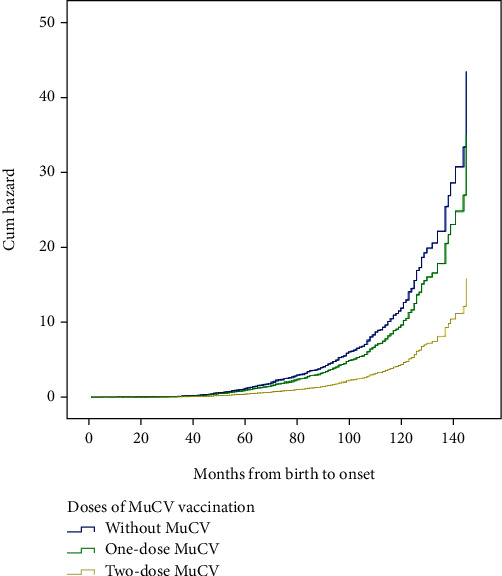
The Cox-proportional hazard curves showing the cumulative hazard with different doses.

The results of Cox-proportional hazard regression analysis adjusted for the covariates are shown in Table 3. The data suggested that onset after SIA, born in 2006, and vaccinated with two-dose MuCV were protective factors against infection even after adjusting for potential confounding effects in the study. For instance, compared with two-dose MuCV, cases without MuCV or with only one-dose MuCV have a higher risk (HR 2.744, 95% CI 2.094-3.597, or HR 2.214, 95% CI 1.812–2.705). However, there was no significant difference in risk between cases without MuCV and those who received one-dose MuCV immunization (*p* = 0.106).  Figure 2 showed the cumulative hazard of mumps with different-dose MuCV by Cox-proportional hazard regression analysis.

## 4. Discussion

In our study, all cases were clinically diagnosed and were mainly characterized by an acute episode of unilateral or bilateral swelling of the parotid gland or other salivary glands. In China, many small hospitals did not have mumps diagnostic reagents and cannot detect mumps immunoglobulin M (IgM) or mumps immunoglobulin G (IgG) titers; isolated mumps virus is even more impossible; the vast majority of clinicians made diagnosis based on the main clinical symptoms. When clinicians encountered infectious diseases in the course of practice, they were mandatorily required to report through CISDCP. A Canadian study showed [9] that detection of IgM presented diagnostic difficulties in a highly vaccinated population, as a demonstrable increase in IgM levels following infection was often delayed or altogether absent in such individuals; detection of mumps virus by reverse-transcription polymerase chain reaction (RT-PCR) was considered the gold standard for mumps diagnosis, but the success of detecting the virus depended on the technique used to collect and transport specimens as well as on the time of collection. An outbreak was reported in New York [10] that a vaccinated student presented with parotitis, IgM testing was negative, and RT-PCR testing was not performed, resulting in a missed diagnosis and the start of an outbreak; RT-PCR was considered the preferred testing method. In China, the detection of mumps virus by RT-PCR was not included in the diagnostic criteria for mumps, which may be an urgent task to modify the diagnostic criteria. Because of the need to control COVID-19, many hospitals have purchased relevant equipment and have the ability to carry out RT-PCR detection. It is feasible to diagnose mumps by RT-PCR in China.

In recent years, mumps outbreaks in vaccinated populations were reported in China and other countries/regions [11]. It is necessary to further study the control measures to control the mumps epidemic. We collected 909 clinical cases of mumps in Quzhou between 2006 and 2018 who were born from 2006 to 2010 and vaccinated with different doses of MuCV. A study in China [12] showed that most of the breakthrough cases of clinically diagnosed mumps are real cases from the analysis of IgM test results sampled in the late stage of onset. However, the reliability of the two methods for verification and diagnosis on the first to seventh days of onset was not high; it was recommended to detect IgM antibody 7 days after onset for verification diagnosis. Except the factor of birth cohort, the mumps incidence after SIA was 1.56 times that before SIA; it may be related to the epidemic age postponed after immunization [13]. The incidence after SIA may be higher if SIA was not implemented. There was no significant difference about the gender of the cases in two periods, and no children were vaccinated with two-dose MuCV in the period before SIA. In the period after SIA, kindergarten children and students accounted for 42.22% and 42.36% of the total cases, respectively. It suggested that special attention should be paid to the vulnerable populations, such as teenagers [14]. Of the 909 cases, the cases without MuCV, with one-dose MuCV, and with two-dose MuCV accounted for 8.14%, 38.94%, and 52.92%, respectively. There was no difference in the sex ratio among the three groups. The mean age of mumps cases was 40 months, 54 months, and 81 months among the different-dose MuCV group. Scattered children without MuCV, kindergarten children with one-dose MuCV, and students with two-dose MuCV were the main cases. It is proved that vaccination with MuCV can change epidemic characteristics of mumps; the peak age of onset shifted backward [15]. Recent mumps outbreaks in individuals who had received two doses of MMR vaccine have challenged the efficacy of the MMR vaccine. However, clinical symptoms, complications, viral shedding, and transmission associated with mumps infection have been shown to be reduced in vaccinated individuals, demonstrating a benefit of this vaccine [16]. There was no significant difference among the cases without MuCV in the 2006-2010 birth cohorts (*χ*^2^ = 8.22, *p* = 0.084); it showed that the sampling was balanced between birth years. Professor Fu et al.'s research [17] showed that the vaccine effectiveness (VE) for one dose of mumps vaccine was 75.0% (95% confidence interval (CI), 33.4–90.6%) to children aged 18 months to 24 months. In the Netherlands [18], mumps outbreaks still occurred with an overall herd immunity threshold of 86–92% and where 96 and 93% received the first and second MMR at 14 months and 9 years, respectively. The SIA targeting children aged 8 months to 4 years of age was implemented in September 2010, which was an action of uniform time, regardless of vaccination intervals and existing antibody titers of mumps. Study [19] showed that the assessment of the mumps antibody titer before vaccination may be a useful complement to vaccination itself, because it is more accurate and cost-effective than direct immunization of unvaccinated subjects.

In order to reduce the interaction between different factors, we used different statistical methods to calculate the cumulative hazard of mumps infection. Kaplan–Meier curves showed that the cumulative hazard of male and female has no difference; it was consistent with the previous conclusion. Kaplan–Meier curves also showed that those who were vaccinated with two-dose MuCV, born in 2006, and infected after SIA detected lower hazards. The results of Cox-proportional hazard regression analysis suggested that onset after SIA, born in 2006, and vaccinated with two-dose MuCV were protective factors against infection even after adjusting for potential confounding effects. The SIA using MM could reduce the number of individuals who failed immunization with MuCV. Adding the second dose of MuCV to the routine immunization schedule or boost immunity may be a strategy to prevent mumps reemergence [20]. The study [21] showed a substantial percentage of subjects lacking a protective mumps titer among medical students who were vaccinated in childhood. Given the higher risk of infection among those subjects, routine preemployment screening should be performed among those operators regardless of their vaccination history and a third dose of MMR should be offered to unprotected students. The protective effect of MMR vaccination was limited for those who had received the MMR dose 13 years or more before infection [22]. Waning immunity was linked to the time since vaccination; result revealed that 72% of confirmed cases received the second dose of MMR ≥ 6 years before symptom onset [23]. These studies further illustrated the importance of strengthening immunization, but when to step up the second dose of MuCV remains to be studied. The immunity waning may account for the higher susceptibility of adolescents and young adults to mumps. It will be modified with the shifting of the second dose of vaccine from two years of age to the preschool age [24]. The average interval between two doses of MuCV in the 2006 birth cohort was 4 years, and it was the longest interval in this study. Timely inoculation of the second dose could provide another opportunity to the children, who might have had primary immune failure or be missing an inoculation. In order to improve the immunity level of the susceptible population and reduce the incidence of mumps, inoculation with two-dose MuCV is necessary for children under 15 years of age [25]. Outbreaks of mumps [26] can occur in schools with high coverage of one-dose MuCV vaccination, the VE of both two-dose and one-dose MuCV wanes over time, the overall VE for two-dose MuCV was superior than that of one-dose MuCV. The VE of one-dose MuCV was 63% for vaccinated within 3 years, 50% for vaccinated within 3 to 5 years, and 34% for vaccinated more than 5 years, and VE for MMR was consistently higher than VE for monovalent mumps vaccine and MM [27]. Professor Li et al.'s research suggested extending the vaccine coverage and providing two-dose MMR for free in China [28].

Therefore, we suggest to carry out a mumps antibody titer persistence study of one-dose MMR in routine immunization in order to find the best time for the second dose of MMR in China. Booster immunization was recommended to control mumps outbreaks in vaccinated populations, but prevaccination screening should be performed to improve the accuracy and cost-effectiveness. There were several limitations in our study. First, all mumps cases were clinically diagnosed without laboratory confirmation. Mumps virus infection can result in symptomatic or asymptomatic infections [29], and clinical parotitis does not have to be mumps virus infection; mumps cases in our study may be underestimated or overestimated. It was necessary to revise the diagnostic criteria of mumps and identify RT-PCR as the standard for mumps diagnosis in China. Second, no serological data were available on the effects of different vaccination intervals; the vaccination interval between two doses needs to be further studied in the future.

## 5. Conclusion

Our study showed that it was necessary to revise the diagnostic criteria of mumps and identify RT-PCR as the standard for mumps diagnosis in China. We suggested that routine immunization schedule should introduce two doses of MMR and prevaccination screening should be performed before booster immunization in vaccinated populations.

## Figures and Tables

**Table 1:** Basic characteristics of mumps among children born from 2006 to 2010 in Quzhou.

Characteristics	Before SIA		After SIA		*χ* ^2^	*ρ* value
	No.	%	No.	%		
All cases	163	17.93	746	82.07	15.21	<0.001
Person-year	299698	23.41	980674.5	76.59		
Gender					0.904	0.342
Male	100	61.35	487	65.28		
Female	63	38.65	259	34.72		
Dose of MuCV					262.52	<0.001
Without MuCV	47	28.83	27	3.62		
One-dose MuCV	116	71.17	238	31.90		
Two-dose MuCV	—	—	481	64.48		
Group classification					144.81	﹤0.001
Scattered children	85	52.15	115	15.42		
Kindergarten children	3	1.84	315	42.22		
Student	75	46.01	316	42.36		
Year of birth^a^					33.467	<.001
2006	82	50.31	143	19.17		
2007	59	36.20	170	22.79		
2008	17	10.43	163	21.85		
2009	4	2.45	146	19.57		
2010	1	0.61	124	16.62		

^a^Cochran's and Mantel-Haenszel (CMH) test was used to analyze the influence of birth cohort to mumps incidence before and after SIA. OR_MH_ = 0.642, 95% CI (0.539-0.766).

**Table 2:** Immunization rates of MuCV by social demographic characteristics among children born from 2006 to 2010 in Quzhou.

Characteristics	Cases without MuCV		Cases with one-dose MuCV		Cases with two-dose MuCV		*χ* ^2^ (*F*)	*ρ* value
	No.	%	No.	%	No.	%		
All cases	74	8.14	354	38.94	481	52.92	—	—
Gender^b^							5.30	0.071
Male	55	9.37	216	36.80	316	53.83		
Female	19	5.90	138	42.86	165	51.24		
Month of onset^a^							168.01	<0.001
Min	1.17		16.72		21.68			
Max	124.52		106.07		151.12			
Average month	39.57 ± 3.59		54.22 ± 1.06		81.30 ± 1.25			
Group classification^b^							183.62	<0.001
Scattered children	44	22.00	93	46.50	63	31.50		
Kindergarten children	20	5.12	204	52.17	167	42.71		
Student	10	3.14	57	17.92	251	78.93		
Year of birth^b^							138.84	<0.001
2006	21	9.33	94	41.78	110	48.89		
2007	18	7.86	75	32.75	136	59.39		
2008	19	10.56	47	26.11	114	63.33		
2009	4	2.67	38	25.33	108	72.00		
2010	12	9.60	100	80.00	13	10.40		

^a^ANOVA test was used to analyze group difference. Mean ± standard deviation. ^b^Chi-square test was used to analyze group differences.

**Table 3:** Results from Cox-proportional hazard regression model depicting the risk of mumps.

Characteristics		*B*	Hazard ratio (HR)	95% CI for HR		*ρ* value
				Lower	Upper	
Gender	Male					
	Female	0.093	1.097	0.956	1.259	0.185
SIA	After SIA					
	Pre-SIA	2.781	16.130	12.078	21.567	<0.001
Year of birth	2006					
	2007	0.784	2.190	1.786	2.684	﹤0.001
	2008	0.929	2.531	2.016	3.178	<0.001
	2009	1.386	4.001	3.133	5.109	<0.001
	2010	1.091	2.978	2.250	3.941	<0.001
Doses of MuCV	Two doses					
	Without	1.010	2.744	2.094	3.597	<0.001
	One dose	0.795	2.214	1.812	2.705	<0.001

## Data Availability

The data used to support the findings of this study are available from the author by email (yzy1815@sohu.com).
